# Actinomycetoma of the arm disseminated to the chest wall

**DOI:** 10.11604/pamj.2015.20.306.5766

**Published:** 2015-03-30

**Authors:** Benhiba Hind, Hassam Badredine

**Affiliations:** 1 Department of Dermatology, Ibn Sina Hospital, Faculty of Medecine and Pharmacy, University Mohamed V- Souissi, Rabat, Morocco

**Keywords:** Actinomycetoma, arm, chest

## Image in medicine

A 27-year-old immigrant sought care for inflammatory nodular swelling lesions over the right arm extending upto the neck with axillary and cervical fistulas lasting for six months (A). His medical history was marked by a long forest stay due to conflicts in his homeland (Mali). A deep biopsy was made for infectious and histopathological examinations. Cultures were negative. Microscopy on hematoxylin and eosin stained sections showed nodular abscesses organized around multilobated grains showing Splendore-Hoeppli Phenomenon (B). Special stains (Gram, Ziehl-Neelsen, Gomori Grocott) were positive. Thus, the histological diagnosis of actinomycetoma probably due to Nocardia was given. In addition, laboratory tests revealed an active hepatitis B infection. After consulting hepatologists, treatment with trimethoprim-sulfametoxazol was introduced, substituted by amoxicillin clavulanate for months with partial clinical response. The evolution was characterized by the reduction of right arm lesions but extension of the infection to the chest wall.

**Figure 1:**
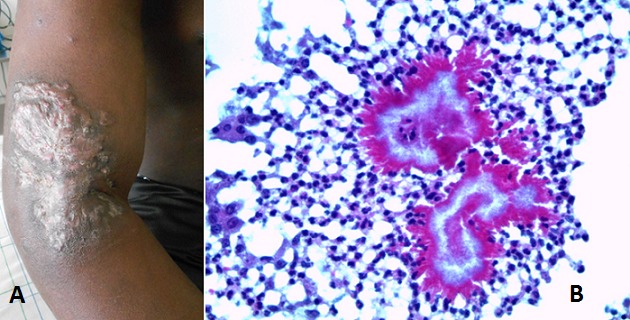
(A) inflammatory nodules of the right arm; (B) cutaneous histology (HE x400): Splendore Hoeppli phenomenon

